# Cardiovascular magnetic resonance imaging in mitral valve disease

**DOI:** 10.1093/eurheartj/ehae801

**Published:** 2024-11-20

**Authors:** Pankaj Garg, Anna Giulia Pavon, Martin Penicka, Seth Uretsky

**Affiliations:** Department of Cardiovascular and Metabolic Health, Norwich Medical School, University of East Anglia, Norwich Research Park, Norwich NR4 7UQ, Norfolk, UK; Cardiology Department, Norfolk and Norwich University Hospitals NHS Foundation Trust, Norwich, Norfolk, UK; Division of Cardiology, Cardiocentro Ticino Institute, Ente Ospedaliero Cantonale, Via Tesserete, 48, 6900 Lugano, Switzerland; Cardiovascular Center, OLV Hospital, Aalst, Belgium; Department of Cardiovascular Medicine, Gagnon Cardiovascular Institute, Morristown Medical Center, 100 Madison Avenue, Morristown, NJ 07960, USA

**Keywords:** Humans, Magnetic resonance imaging, Mitral valve, Mitral valve insufficiency, Papillary muscles, Heart valve diseases, Chordae tendineae, Haemodynamics, Heart failure

## Abstract

This paper describes the role of cardiovascular magnetic resonance (CMR) imaging in assessing patients with mitral valve disease. Mitral regurgitation (MR) is one of the most prevalent valvular heart diseases. It often progresses without significant symptoms, leading to left ventricular overload, dysfunction, frequent decompensated heart failure episodes, and excess mortality. Cardiovascular magnetic resonance assessment is recommended for MR when routine ultrasound imaging information is insufficient or discordant. A well-planned CMR can provide an in-depth assessment of the mitral valve apparatus, leaflet morphology, and papillary muscles. In addition, it can precisely inform the impact of MR on left atrial and ventricular remodelling. The review aims to highlight established and emerging techniques for morphological assessment, flow assessment (including regurgitation and stenosis), myocardial assessment, and haemodynamic assessment of mitral valve disease by CMR. It also proposes a simplified clinical flow chart for CMR assessment of the mitral valve.

## Graphical Abstract

**Graphical Abstract:**
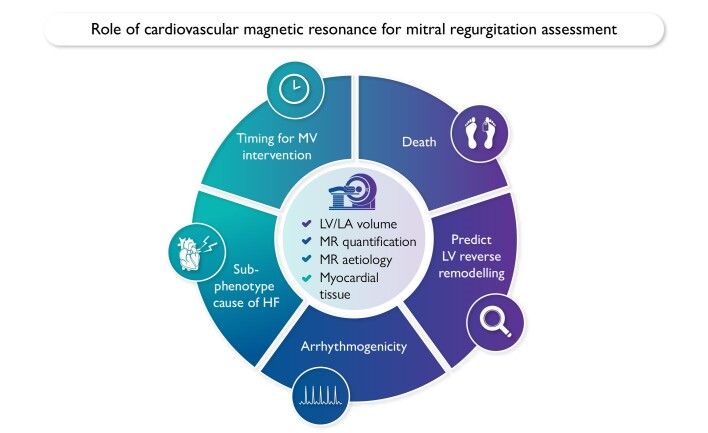
Role of cardiovascular magnetic resonance in mitral regurgitation (MR) assessment, focusing on left ventricular/left atrial (LV/LA) volumes, MR quantification, aetiology, and myocardial tissue. Key implications include predicting LV remodelling, timing of MV intervention, identifying heart failure sub-phenotypes, assessing arrhythmogenicity, and mortality risk.

## Introduction

Cardiovascular magnetic resonance (CMR) imaging has emerged as an important test for the assessment of patients with mitral valve (MV) disease. Mitral regurgitation (MR) is one of the most common valvular heart diseases (VHD) worldwide, and its prevalence increases with age, reaching 13.3% in individuals aged 75 years and older.^1^ Mitral regurgitation can progress without significant symptoms, leading to left ventricular (LV) overload and dysfunction. Hence, MR is associated with excess mortality and frequent decompensated heart failure (HF) episodes. The European Society of Cardiology (ESC) and the American College of Cardiology/American Heart Association guidelines recommend CMR assessment in VHD when insufficient or discordant information is obtained from an echocardiogram.^2,3^ A well-planned CMR can provide an in-depth assessment of the MV apparatus, leaflet morphology, papillary muscles, and its impact on the left atrium and left ventricle by the evaluation of their size, function, and myocardial fibrosis or scar burden (SB).

In this state-of-the-art review, we aim to highlight established and emerging techniques for morphological assessment, flow assessment, including regurgitation and stenosis, and haemodynamic assessment of MV disease by CMR. This review will recommend a simplified clinical flow chart for CMR assessment of the MV (*Graphical Abstract*).

## Mitral valve anatomy—mitral regurgitation mechanism

**Figure 1:**
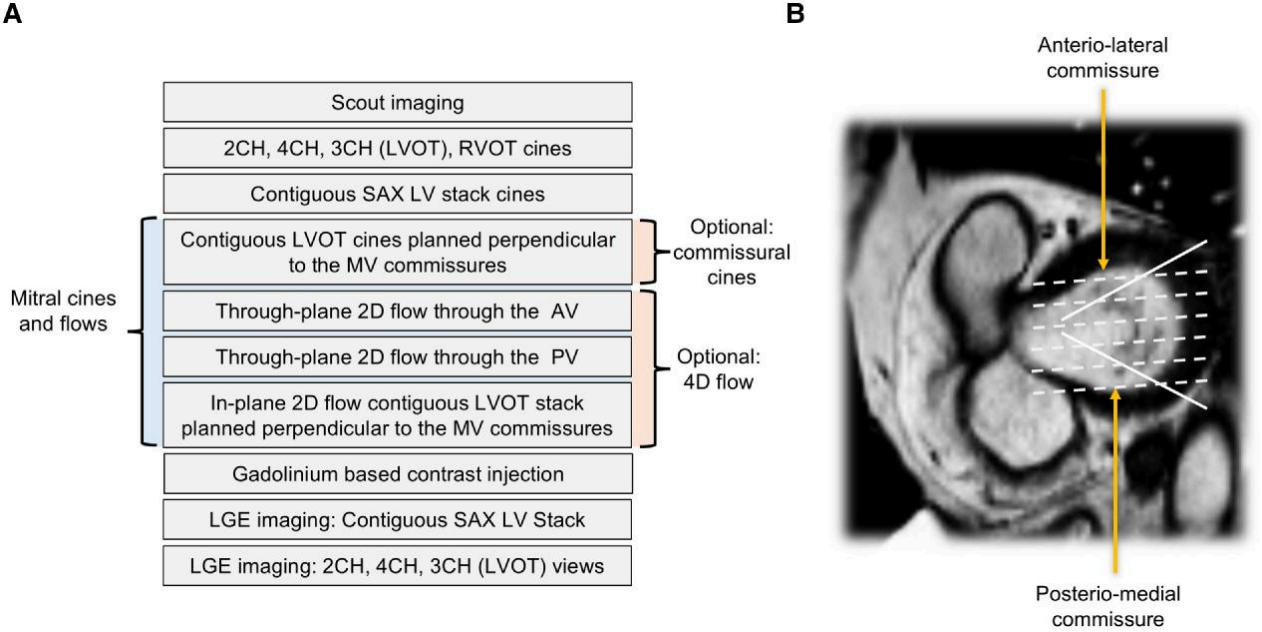
(*A*) Recommended CMR protocol for mitral valve assessment by CMR. (*B*) How to plan the long-axis cine acquisitions of the mitral valve using the enface short-axis mitral valve image. Contiguous modified three-chamber cines intersecting the commissural line are planned to localize segmental pathologies such as billowing, prolapse, flail, thickening, or calcification (*B*: dotted white lines). When the commissures are at an angle, additional commissural cines may be planned (*B*: solid white lines)

Any structural or functional impairment of the MV apparatus that exhausts MV tissue reserve available for leaflet coaptation will result in MR. Cine imaging is the most widely used CMR visual assessment method for valve anatomy and motion.^4,5^ A dedicated CMR protocol for MV disease is detailed in *Figure 1*.

**Figure 2:**
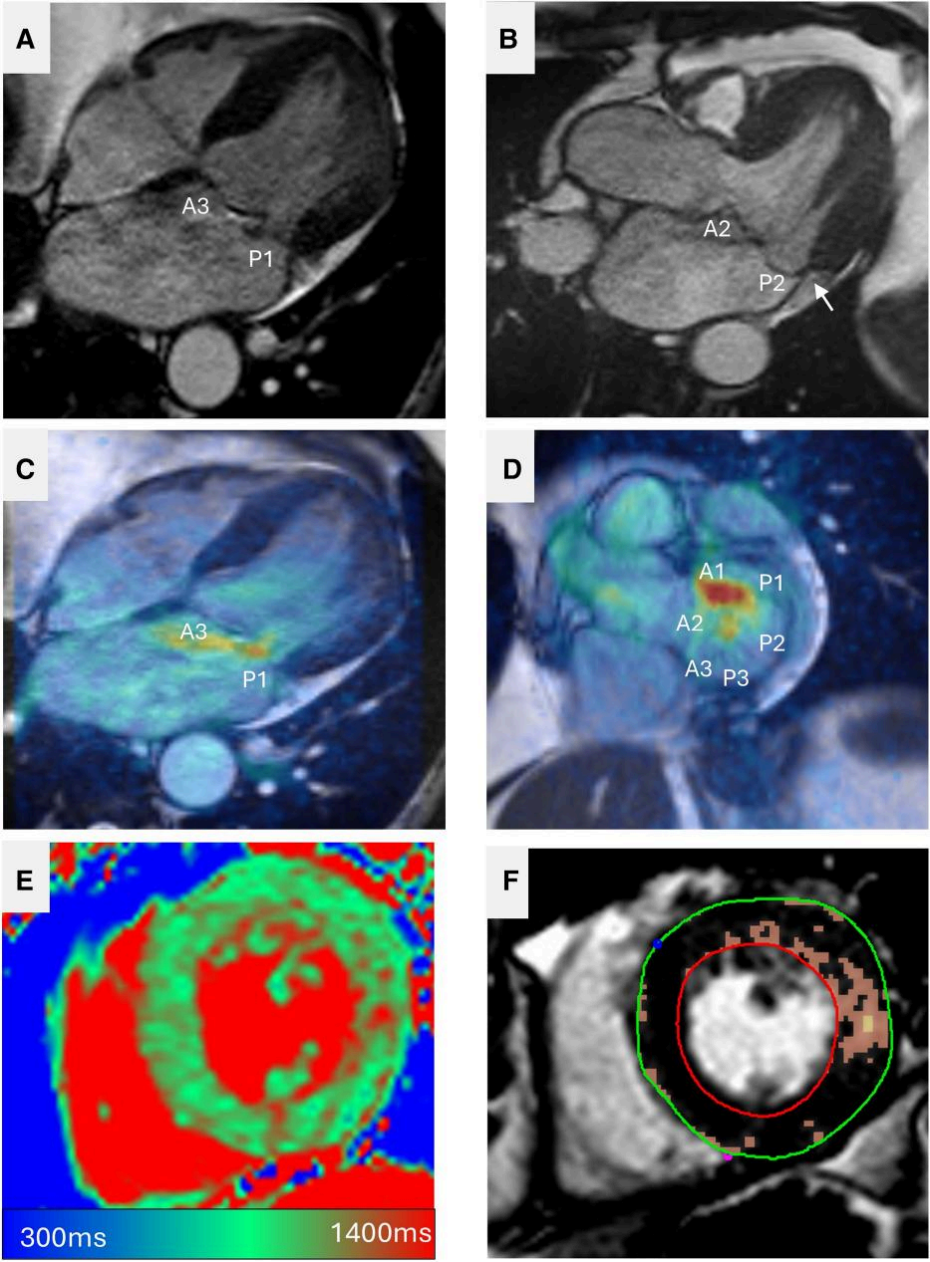
CMR assessment of MVP morphology and associated myocardial tissue changes. (*A* and *B*) In the three-chamber cines, it is evident that there is a P1 prolapse resulting in an MR jet directed towards the intra-atrial septum. It is also clear that the left atrium is dilated. The white arrow in *B* highlights MAD. (*C* and *D*) Velocity superimposition on cines using 4D flow CMR to highlight MR jet. In *D*, it is evident that the MR jet originates at P1/P2 level. (*E* and *F*) Myocardial tissue characterization reveals higher native T1 values and scar/fibrosis in the lateral wall on LGE imaging

**Figure 3:**
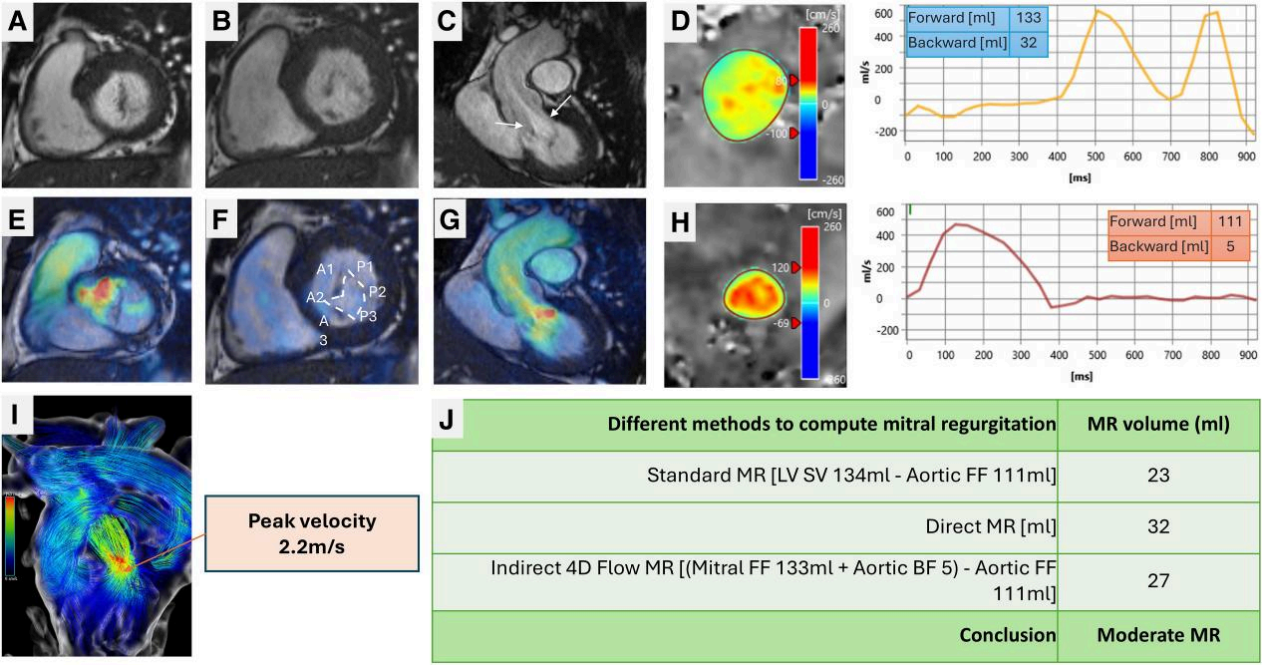
Primary MR with congenital cleft mitral valve and normal mitral valve leaflet motion (Carpentier I). (*A–C*) In both short-axis and long-axis cines, at the level of the A2 leaflet, the anterior mitral valve leaflet is split but connected in a V-shape with a raphe in between. This raphe (white arrows) is seen in the LV outflow tract. (*D*) Mitral flow quantification using 4D flow and compensating for myocardial motion by using valve tracking. (*E–G*) 4D flow superimposed velocity demonstrates flow acceleration in the LV outflow tract during systole. Also, eccentric MR is seen, which is directed towards the lateral LA wall. (*H*) Aortic flow mapping using 4D flow data. (*I*) 4D flow streamlines in three-dimension demonstrate the flow acceleration in the LV outflow tract due to the MV raphe with a 2.2 m/s peak velocity. (*J*) Standard and two 4D flow methods to quantify MR demonstrate that it is at most moderate MR only

The most common cause of primary MR is degenerative MV disease, leading to predominately mitral valve prolapse (MVP) and/or flail leaflet due to chordal rupture (*Figure 2*). Cine imaging helps to recognize and diagnose MVP on cines in both short- and long-axis views. Other causes of primary MR include rheumatic heart disease-associated MV leaflet thickening, calcification, and sub-valvular changes. There is a growing body of evidence that cine CMR can also help diagnose and describe these pathological changes.^6–8^ However, the limited spatial resolution of CMR, with a slice thickness of 5–6 mm, hinders the detailed visualization of the MV tip, which usually has a 1–5 mm thickness, thus making the differentiation between segment prolapse and flail less accurate.^9^ This limitation of CMR is especially relevant in cases where infective endocarditis of the MV is suspected to be the cause of primary MR. Other causes of primary MR include congenital conditions such as an isolated cleft, a double orifice, or a parachute MV (*Figure 3*). These conditions can also be identified by cine CMR.

**Figure 4:**
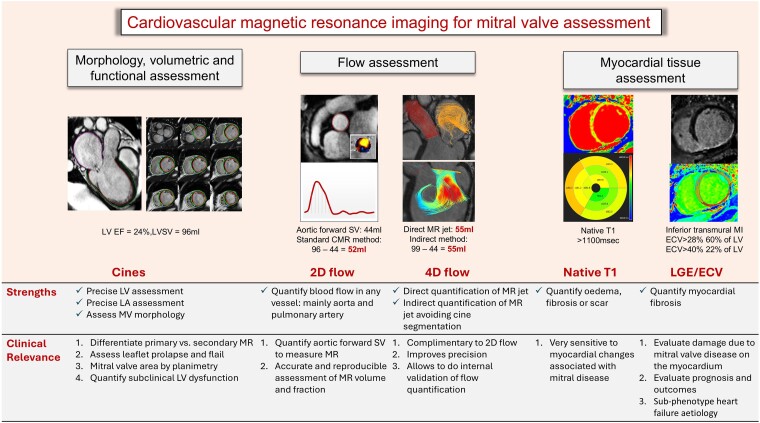
Role of CMR in ischaemic MR. This is a case of severe secondary MR and extensive transmural inferior myocardial infarction. This patient had a high degree of VT burden and needed an intra-cardiac defibrillator. LV filling pressure was high using LA volume and LV mass.^13^

Secondary MR or functional MR stems from maladaptive LV or left atrial (LA) remodelling that leads to the maladaptation of intrinsically normal mitral leaflets. Secondary MR is the leading cause of VHD that leads to HF, arrhythmia, and death.^10^ While secondary MR broadly refers to MR stemming from adverse LV or LA remodelling, the mechanism for this varies. In ischaemic cardiomyopathy, localized myocardial injury (ischaemia or infarction) in regions underlying the MV can result in MV tethering and insufficiency (*Figure 4*). In non-ischaemic cardiomyopathy, diffuse myocardial fibrosis augments LV stiffness, increasing mitral afterload and contributing to secondary MR. More recently, evidence has emerged suggesting functional MR can also be caused by LA enlargement. This is mechanistically associated with annular dilatation, perturbations of annular contraction and leaflet tethering.^11^ Myopathic contributors to secondary MR can also be mixed, with both infarction and non-ischaemic fibrosis coexisting. Given the differences in mechanism and imprecision of clinical assessment, myocardial tissue characterization imaging is well suited to improve clinical decision-making and prognostic outcomes for patients with secondary MR. The 2023 ESC Guidelines for managing cardiomyopathies highlight the role of CMR in assessing the aetiology, function, and prognosis of ischaemic and non-ischaemic cardiomyopathy.^12^

## Quantification of mitral regurgitation

Echocardiography remains the first-line test for the diagnosis or screening for MR. This is mainly because echocardiography is versatile, portable, quick, readily available, and cost-effective. Nevertheless, current recommendations rely on several echocardiographic parameters [proximal isovelocity surface area (PISA), and vena contracta] to make an assessment of MR severity.^14^ Due to the discordance in MR grading between these parameters,^15,16^ the guidelines recommend a hierarchy-based algorithm to facilitate the grading of MR severity.^14^ There is also a clinical need for a complementary imaging modality that offers MR quantification where echocardiography may not be ideal. Cardiovascular magnetic resonance uses precise quantification of LV volumes by cines and flows by two-dimensional (2D) flow imaging which uses the phase-contrast velocity-encoded imaging to quantify MR. Furthermore, the standard methods of CMR quantification of MR do not depend on MR jet characteristics.

In real-world practice, both imaging modalities can complement each other in evaluating MV disease. Echocardiography is live imaging, whereas CMR is averaged imaging. Live imaging can be powerful for elucidating beat-by-beat assessment but can also result in higher variability in MR assessment. Meanwhile, averaged imaging introduces better reproducibility but cannot provide temporal information similar to live imaging. Inherent differences in acquisition and post-processing can result in differences between CMR and echocardiography quantification of MR^17–23^ (*Table 1*). There are several reasons for this variation. Firstly, echocardiography often provides lower ventricular volume measurements compared with CMR. This difference arises due to factors such as echocardiography’s reliance on geometric assumptions for volume calculations, challenges in endocardial border delineation, and the potential for foreshortening of the ventricles.^24,25^ Secondly, for aortic stroke volume (SV), echocardiography requires assumptions about the LV outflow tract area, which can sometimes lead to lower forward SV measurements.^26^ Some of these limitations can be reduced by using contrast-enhanced or three-dimensional echocardiographic acquisitions. Both have been shown to improve agreement with CMR for LV volumetric assessment.^24,27^ Similar to echocardiography, CMR has its technical issues. Firstly, in patients with arrhythmias, CMR acquisitions can have significant temporal blurring that can result in underestimating the LV SV. Secondly, phase errors, including background and offset errors, can further introduce inconsistencies in flow quantification. Finally, not every patient is suitable for CMR examination as ∼1% of patients have claustrophobia,^28^ and patients with implantable cardioverter–defibrillators will have significant shadowing in cine imaging,^29^ prohibiting accurate LV SV quantification. These technical issues with echocardiography and CMR highlight why it is clinically important to have a multi-modality approach in the assessment of MV disease.

**Table 1:** Studies comparing CMR and echocardiography in the assessment of MR

Study	Year	*N*	Echo method	Absolute agreement	Agreement if severe	Severe MR	
						Echo	CMR
Cawley *et al*.^17^	2013	26	PISA	13/23 (57%)	5/12 (42%)		
Uretsky *et al*.^18^	2015	103	PISA	27/103 (36%)	13/60 (22%)	58	15
Lopez-Mattei *et al*.^19^	2016	70	Doppler Vol	44/70 (63%)	2/10 (20%)	8	2
Sachdev *et al*.^20^	2016	50	PISA	23/50 (46%)	10/15 (66%)	14	11
Penicka *et al*.^21^	2017	258	PISA		62/123 (50%)	100	85
Uretsky *et al*.^22^	2022	152	PISA/ASE algorithm	79/152 (52%)	32/82 (39%)	79	32
Altes *et al*.^23^	2022	188	PISA	109/188 (58%)	61/140 (43%)	121	80
Altes *et al*.^23^	2022	188	Doppler RVol	100/188 (53%)	41/110 (37%)	71	80

ASE, American Society of Echocardiography; PISA, proximal isovelocity surface area; RVol, regurgitant volume.

**Figure 5:**
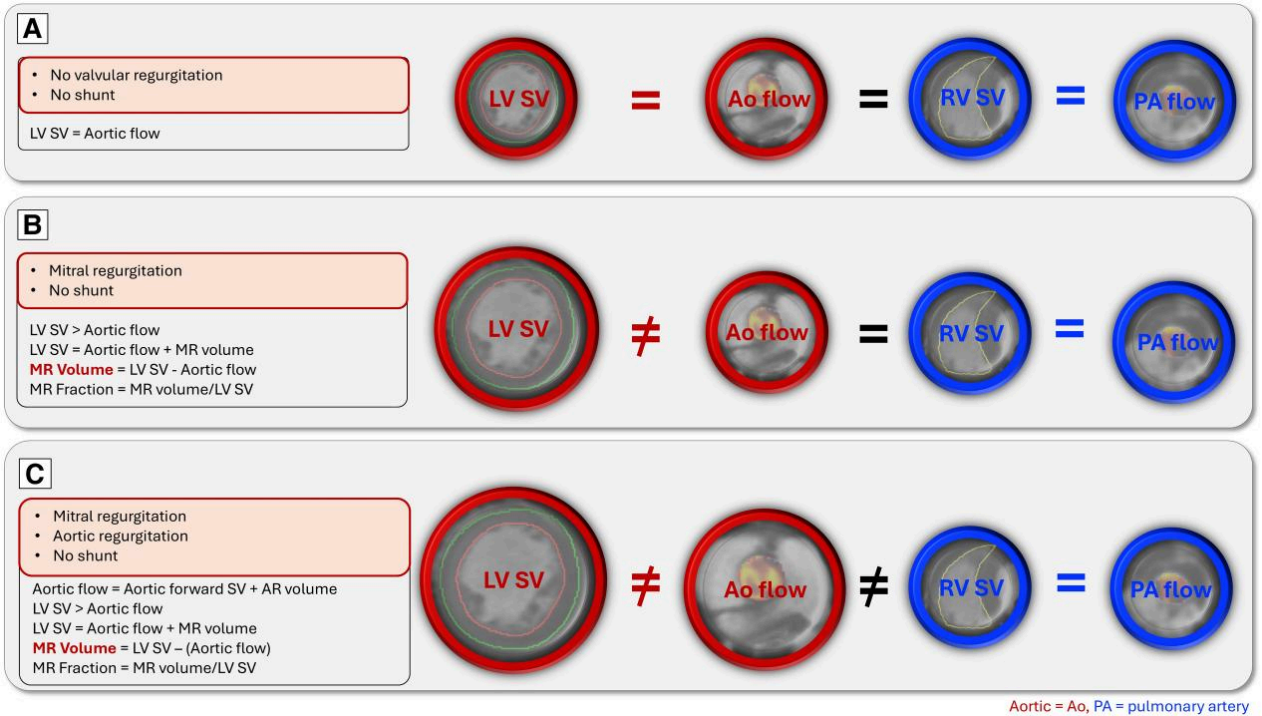
Standard MR quantification methods using CMR. (*A*) In the patient without VHD or a cardiac shunt, the ventricular SVs, aortic flow, and PA flow are all equal. (*B*) In the patient with MR and no cardiac shunt, the LV SV is increased due to the presence of MR. The RV SV, aortic flow, and PA flow all equal each other. The MR volume is calculated as LV SV − aortic flow. (*C*) In the patient with MR and AR and no cardiac shunt, the LV SV is increased due to the presence of MR and AR. Aortic flow now includes aortic forward SV and AR. AR volume can be measured directly using the diastolic flow of the aortic flow

In CMR, MR can be quantified by multiple methods, which allows the investigation of the conservation of mass principle applied to flow. This provides a ‘check and balances’ system when quantifying forward SV, allowing internal validation on flows and ensuring that the regurgitation volume is accurate (*Figure 5*, Supplementary data online, *Video S1*).

The overarching equation of conservation of mass principle is as follows:

**LV SV** by cine is equal to the following:

- **LV inflows** (mitral inflow SV + aortic regurgitation [AR]),
- **LV outflows** (aortic forward SV + MR),
- **Right ventricular (RV) SV** by cine,
- **RV outflows** [pulmonary artery (PA) forward SV + tricuspid regurgitation].

Application of this allows us to quantify MR by standard cine and phase-contrast flow CMR in several following ways:

1. **Standard method** (no cardiac shunt, MR, no AR): The first step to quantify MR is to do an LV volumetric assessment. The endocardial border of the left ventricle is manually or semi-automatically contoured on each short-axis slice at both end-diastole and end-systole to compute end-diastolic volume and end-systolic volume. Subtracting end-systolic volume from end-diastolic volume equals LV SV. In MR, the LV SV is increased as it includes the aortic forward flow SV and the MR volume. Mitral regurgitation volume is calculated as LV SV−aortic forward flow SV, and MR fraction (MRF) is calculated as MR volume/LV SV. In cases where aortic forward flow SV could suffer from flow acceleration due to aortic stenosis or LV outflow tract obstruction, other SVs could be used (RV SV or PA forward flow SV).
2. **In cases of MR and AR** (no cardiac shunt): In these patients, the LV SV includes the aortic forward flow SV, the AR volume, and the MR volume. Aortic regurgitation can be quantified directly from the aortic diastolic flow mapping, and the MR volume can be calculated as LV SV−(aortic forward flow SV + AR volume).

In the first method, the standard method, the aortic forward SV, can be replaced by the RV SV or the PA forward SV to check if all results are in agreement for MR quantification. It allows the detection of errors in either segmentation of the RV SV, acquisition of the aortic or pulmonary flows, or the presence of aortic stenosis, pulmonic stenosis, or LV outflow tract obstruction, which affects the accuracy of flows. In patients with MVP, it remains controversial if the basal line for the left ventricle at end-systole should be placed at the top of the left ventricle or the level of the leaflets.^30,31^ Grading of MR severity by CMR is detailed in *Table 2*.

**Table 2:** Grading MR severity by CMR

		Grading of MR severity		
Type of MR		Mild	Moderate	Severe
Primary^21,32^	MRF	<20%	20%–39%	>40%
	MR volume	<30 mL	30–55 mL	>55–60 mL
Secondary^33,34^	MRF	<20%	20–34%	≥35%
	MR volume	<30 mL	30–55 mL	>55–60 mL

### Four-dimensional flow CMR

**Figure 6:**
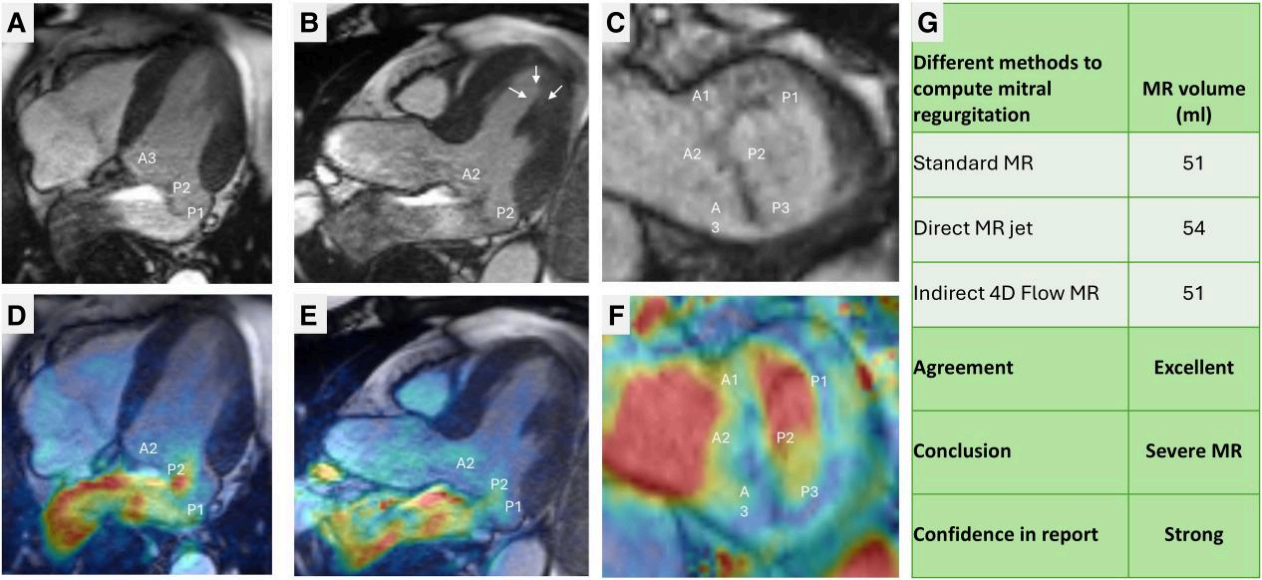
Primary MR quantification (Carpentier type II). (*A–C*) Morphological assessment of the mitral valve is first made on cines. In these first three panels, it is evident that there is both anterior and posterior MVP, resulting in the dooming of both leaflets. In *C*, which is an extension of the short-axis LV volumetric assessment, it is clear that the prolapse is worse at the P1 and P2 levels. (*D–F*) On 4D flow velocity superimposition on top of cines, it is clear that the prolapse is resulting in an eccentric MR jet directed towards the intra-atrial septum. This jet is seen in *E* to cause flow reversal in upper pulmonary veins. (*G*) Three main methods of MR have been used to quantify MR, and they have excellent agreement between them. This builds confidence in reporting the degree of MR, which in this particular case is severe

Four-dimensional flow (4D flow) CMR is emerging as a valuable tool for the evaluation of MR.^35^ It provides some enhancements over the traditional 2D flow method.^36^ The benefits of using 4D flow CMR for MR quantification are manifold. It allows for a single acquisition, single sequence, and retrospective analysis. Most importantly, it allows MR visualization in both 2D and three-dimensional—which adds qualitative assessment, especially when CMR findings are being presented in the multidisciplinary heart meeting to facilitate clinical decisions (*Figure 6*, Supplementary data online, *Video S2*). When the velocity data are superimposed on cine images, it can further allow the assessment of the number of MR jets, the eccentricity of the MR jet, and the duration of the MR jet in the left atrium. Four-dimensional flow enables valve tracking to account for motion throughout the cardiac cycle and directly measures MR. The use of single acquisition improves the precision, as one can quantify all four valvular flows for the same averaged cardiac cycles.^37,38^ This unique advantage has led 4D flow to be defined as the reference method for intra-cardiac transvalvular flow assessment.^39^ This reduces room for error when comparing different valvular flows. This direct quantification of the regurgitant jet proves especially beneficial in multiple valvular pathologies or patients with intra-cardiac shunts such as ventricular septal defects. In these scenarios, standard methods described earlier are not feasible due to the shunting volume.

For 4D flow, a retrospectively electrocardiogram-gated sequence covering the complete cardiac cycle, with a temporal resolution of at least 45 ms and a spatial resolution of 3 mm × 3 mm × 3 mm or better, is recommended.^39^ The field of view should preferably cover the whole LV, LA, and aortic outflow tract, including the proximal ascending aorta. Prior to analysis, 4D velocity data should be carefully checked for errors, and where possible, these errors should be resolved.

**Figure 7:**
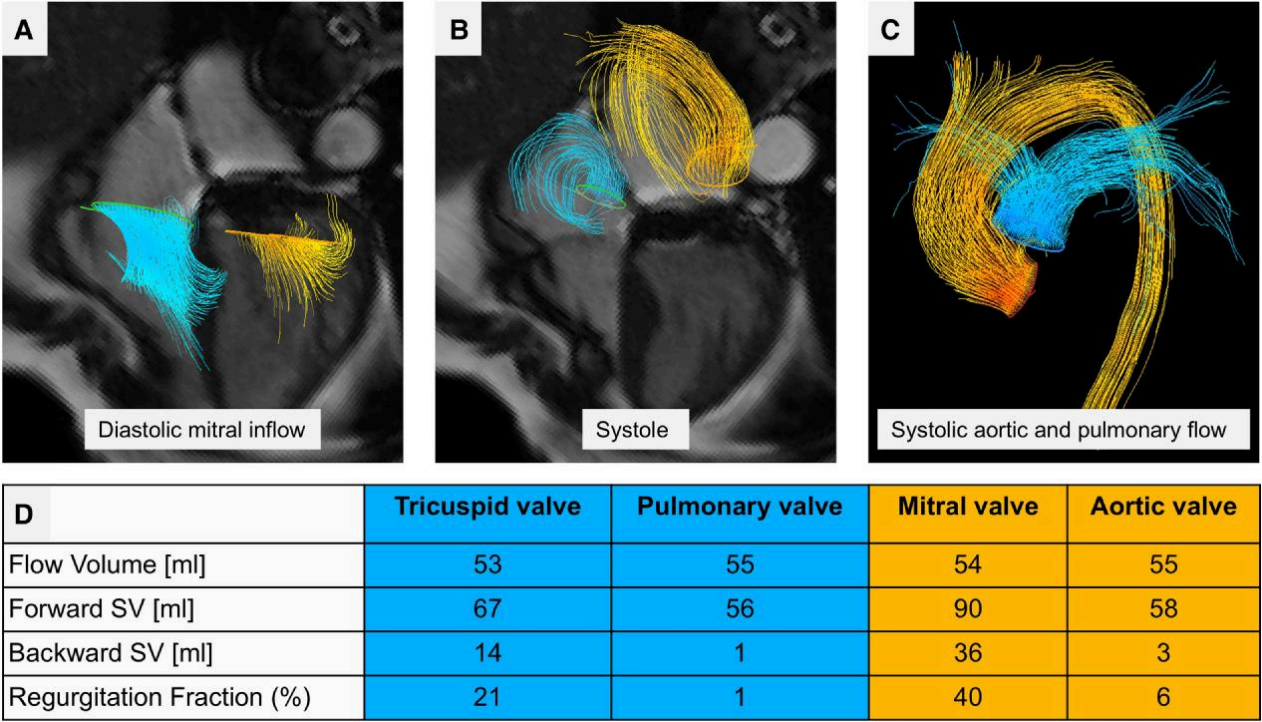
Four-dimensional flow assessment in a patient with mitral valve replacement. (*A*) Diastolic mitral inflow streamlines. For mitral inflow, the reconstruction plane is below the MVR to avoid the artefacts due to it. (*B*) Both mitral and tricuspid regurgitation planes are tracked throughout the ventricular systole. (*C*) Both pulmonary and aortic flows are quantified to apply the conservation of mass principle to flow. (*D*) The flow volume across all four valves is in excellent agreement: this builds confidence in the MR regurgitation fraction (40%). In this case, the MR is severe, and the TR is mild

Using 4D flow, several methods can be applied to quantify MR. Firstly, very similar to the standard method, 4D flow can be used to quantify aortic forward flow SV and subtracted from LV SV to quantify MR. Direct MR jet tracking during systolic phases is also possible, but it can prove challenging in cases with dynamic eccentric primary MR. Finally, by tracking the MV, mitral inflow SV and backward flows can be quantified on top of aortic flows. By applying the conservation of mass principle, MR volume can be calculated using the following equation: MR volume (mL) = (mitral inflow SV + AR) − aortic forward flow SV. This approach has been shown to have the highest degree of intra-/inter-operability in all types of MR, including primary and secondary MR and even in patients with MV replacement^37^ (*Figure 7*). Hence, 4D flow is complementary to established 2D flows and provides an enhanced visual and quantitative assessment of MR or even mitral stenosis (MS).

## Consequences on the ventricle and left atrium

Mitral regurgitation imposes significant haemodynamic effects on the left atrium and left ventricle. Mitral regurgitation is primarily a volume overload to the left ventricle.^40^ The primary compensatory response of the left ventricle to MR is dilatation, primarily an increase in LV end-diastolic volume. This remodelling can further exacerbate MR by disrupting the normal function of the MV apparatus. A strong correlation has been observed between MR volume quantified by CMR and indexed LV end-diastolic volume, with increasing indexed LV end-diastolic volume associated with increasing MR volume.^40^ Studies have shown that MV intervention resulting in decreased MR will allow the left ventricle to reverse remodel with a decrease in LV end-diastolic volume.^41^ In a prospective multicentre study of CMR vs. echocardiography, assessing the concordance, inter-observer variability, and response of the left ventricle to surgery showed not only significant discordance between echocardiography and CMR for MR quantification but also MR volume quantified by CMR was strongly correlated to post-surgical LV reverse remodelling (*r* = .85, *P* < .0001), whereas this was not the case for echocardiography (*r* = .32, *P* = .1).^18^ Another study assessed the newest American Society of Echocardiography algorithm against CMR.^22^ The authors found that only CMR-based regurgitant volume and fraction were independent predictors of post-surgical LV reverse remodelling, and severe MR by echocardiography was not a predictor of post-surgical LV remodelling. In another multicentre study, Uretsky *et al*.^42^ found that CMR-quantified MR can be used to predict the degree of reverse remodelling and the size of the LV end-diastolic volume post-surgery, further highlighting the accuracy of CMR-quantified MR.

One of the strengths of CMR is the ability to characterize the myocardium using both traditional late gadolinium enhancement (LGE) techniques and newer T1 mapping techniques (*Figure 4*). Beaufils *et al*.^43^ studied 400 patients with MVP and reported the presence of LGE in 28% of patients. In this study, the presence of LGE was associated with increased arrhythmias and cardiovascular events. Similarly, Kitkungvan *et al*.^44^ found an LGE present in 37% of patients with MVP but only 7% of patients without MVP. Late gadolinium enhancement was associated with greater MR volume and fraction and lower LV ejection fraction.

Increased myocardial fibrosis, either due to primary MR itself or other concomitant diseases, could be an important marker in patients who warrant earlier referral to MV intervention. Liu *et al.*^45^ showed that among patients with primary MR referred for MV surgery, there is increased myocardial collagen deposition on myocardial biopsy among patients with asymptomatic and symptomatic MR compared with controls. Importantly, the fibrosis present in patients with MR in this study was diffuse and thus more likely detected with T1 mapping extracellular volume (ECV) than LGE. In a small study of 35 patients, Edwards *et al*.^46^ found that increased T1 mapping-based ECV was associated with reduced exercise capacity in patients with asymptomatic MR. A second study by Kitkungvan *et al*.^47^ found that T1 mapping-based ECV was predictive of the need for MV surgery and was able to stratify which patients with MRF ≥40% would decompensate.

Based on the existing data, CMR’s ability to characterize myocardial fibrosis using either LGE or T1 mapping techniques may be useful in helping determine which patients with MR have a worse prognosis. Whether this will help determine which patients may benefit from earlier surgery and which will benefit from watchful waiting needs further study.

In the context of the left atrium, MR can lead to LA enlargement and dysfunction.^48^ The regurgitant flow from the left ventricle to the left atrium during systole increases LA volume and pressure, leading to LA remodelling and dysfunction. Cardiovascular magnetic resonance plays a significant role in assessing LA volume and function, and it can accurately measure the effect of acute and dynamic changes in preloading conditions on the left ventricle.^49^ More importantly, recent evidence suggests that CMR can also measure LV filling pressure, offering a more integrated haemodynamic assessment.^50^

## Clinical outcomes

The prognostic role of echocardiography in MR assessment is well established.^51,52^ More recently, several studies have evaluated the prognostic complementary role of CMR in MR assessment^21,32–34,53^ (*Table 3*).

**Table 3:** Clinical outcome and prognostic studies for MR assessment by CMR

Study	Year	Patients (*N*)	MR grading	HR (95% CI)	*P*	Clinical outcome
Myerson *et al*.^32a^	2016	109	RVol ≤55 mL MRF ≤40%	.81 (.72–.88) .79 (.70–.86)	<.01 <.01	Symptoms Onset MVR indication
Penicka *et al*.^21a^	2018	258	LVESVi per 10 mL/m^2^	1.40 (1.05–1.81)	.04	All-cause mortality
				1.30 (1.10–1.56)	.03	MVR indication
				1.30 (1.05–1.62)	.04	Mortality and MVR
			RVol per 10 mL	1.10 (1.05–1.20)	.03	All-cause mortality
				1.23 (1.06–1.29)	<.01	MVR indication
				1.20 (1.05–1.30)	<.01	Mortality and MVR
			MR category	1.64 (1.21–1.83)	<.01	All-cause mortality
				1.79 (1.58–1.91)	<.01	MVR indication
				1.76 (1.63–1.85)	<.01	Mortality and MVR
Tayal *et al*.^53b^	2021	441	MRF ≥ 30%			
		139 No SB		2.17 (.62–7.62)	NS	All-cause mortality Heart Transplant
		109 SB (< 4%)		.86 (.33–2.19)	NS	
		100 SB (<20%)		3.10 (1.39–6.89)	<.01	
		93 SB (≥ 20%)		1.39 (.53–3.69)	NS	
Cavalcante *et al*.^33b^	2020	578	MRF ≥ 35%			
		SB < 15%		.95 (.41–2.21)	<.01	All-cause mortality Heart Transplant
		SB15–29%		.98 (.21–4.59)	<.01	
		SB ≥ 30%		2.86 (1.78–4.57)	<.01	
Wang *et al*.^34b^	2023	1414	MRF ≥ 35%			
Ischaemic CM		SB ≥ 5%		1.87 (1.25–2.81)	<.01	All-cause mortality Heart Transplant
Non-ischaemic CM		SB ≥ 2%		2.67 (1.26–5.63)	<.01	

CM, cardiomyopathy; ESVi, end-systolic volume indexed; LV, left ventricle; MR, mitral regurgitation; MRF, mitral regurgitant fraction; MVR, mitral valve replacement surgery; RVol, regurgitant volume; SB, scar burden.

^a^Organic MR.

^b^Functional MR.

Myerson *et al*.^32^ performed a multicentre prospective study of 109 asymptomatic patients with moderate or severe primary MR initially assessed by echocardiography. Following CMR evaluation, patients were monitored for a mean period of 2.5 ± 1.9 years to determine the need for surgery. The study found that a CMR-derived MR volume >55 mL was the most accurate predictor for indicating MR surgery (*P* < .0001). Additional CMR metrics, such as MR volume index and MRF, were also significant predictors. These results suggest that while echocardiography provides crucial initial assessment, CMR could offer enhanced prognostic accuracy, particularly in determining surgical intervention needs.

A second prospective study was performed by Penicka *et al*.,^21^ including 258 asymptomatic patients with moderate or severe MR by initial echocardiography assessment. Over a median 5-year follow-up, CMR demonstrated low intra- and inter-observer variability, enhancing its reliability in measuring MR severity. The study found that CMR-derived MR volume had a higher area under the curve for predicting all-cause mortality (.72 vs. .61) and the need for mitral valve surgery (.77 vs. .63). Importantly, CMR accurately predicted clinical outcomes when CMR and echocardiography results were discordant. These studies highlight that CMR offers enhanced precision in ambiguous cases, ensuring a more comprehensive evaluation. By integrating both modalities, clinicians can better align imaging findings with patient symptoms, leading to more appropriate management decisions.

**Figure 8:**
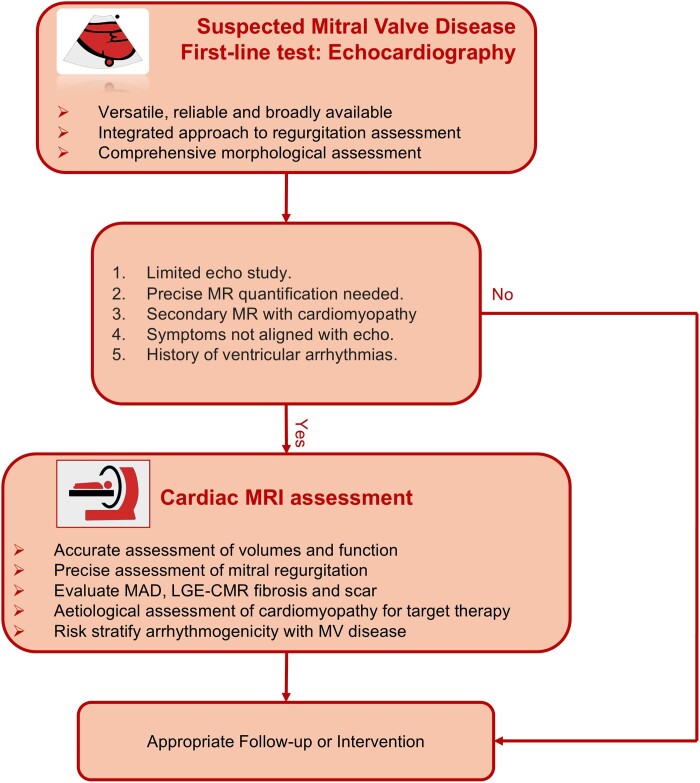
Recommended clinical pathway for the use of CMR for mitral valve disease assessment

Late gadolinium enhancement-measured focal non-ischaemic fibrosis is known to occur with secondary MR and increases with LV dilatation.^54,55^ A study by Tayal *et al*.,^53^ involving 441 patients with secondary MR, found that an MRF ≥30% significantly increased the risk of adverse events, including all-cause mortality or heart transplant, in patients with LV scar. This heightened risk was absent in patients without LV scar. Moreover, mortality in secondary MR increases at lower thresholds of MR due to myocardial injury-related scar.^33,56^ A 2009 study highlighted that the severity of posterior papillary muscle scarring correlates with decreased segmental wall motion and poorer MR correction following coronary revascularization and annuloplasty. This suggests that routinely assessing SB may help identify patients for whom annuloplasty alone cannot eliminate MR.^57^ Cavalcante *et al*. recently showed how not only quantification of secondary MR by CMR can stratify the risk but also the interaction between MR quantification and SB can provide further risk stratification beyond LV volumes and clinical parameters. On top of that, it was possible to further classify patients with significant secondary MR (MRF > 35%) and large SB (>30% of LV mass), carrying a very high risk for all-cause mortality and/or heart transplant, despite surgical MV intervention. In comparison, patients with low SB (<15% of LV mass) had survival benefits if surgically treated.^33^ Finally, a large observational study which recruited 1414 patients undergoing CMR for cardiomyopathy (ejection fraction <50%) assessment demonstrated that LGE played a critical, independent role in prognostication on top of secondary MR quantification.^34^ The primary outcome in Wang *et al*. study was all-cause death, heart transplant, or LV assist device implantation during follow-up. The optimal MRF threshold for moderate and severe secondary MR was ≥20% and ≥35%, respectively, in both ischaemic and non-ischaemic cardiomyopathy, based on the prediction of the primary outcome. Similarly, optimal LGE thresholds were ≥5% in ischaemic cardiomyopathy and ≥2% in non-ischaemic cardiomyopathy to predict the primary outcome. The observations of these studies demonstrate that CMR is an important non-invasive imaging modality to personalize treatment decisions, and myocardial tissue characteristics should be incorporated in the assessment of MR (*Figure 8*).

## Limitations of CMR assessment for MV disease

While CMR imaging is valuable for MV disease assessment, it has limitations. These include susceptibility to arrhythmia artefacts, acceleration methods that can reduce temporal resolution, which can result in overestimation of LV end-systolic volume, and challenges posed by patient factors such as claustrophobia and metallic implants. Short-axis cine segmentation for LV volumetric assessment can also be inaccurate due to spatial misalignment between slices and due to variability in basal slice segmentation. These issues with short-axis cine segmentation can introduce errors in MR quantification. Moreover, aortic forward SV can be underestimated in non-laminar aortic flows which also result in significant overestimation of MR volume. In MS, 2D flow can underestimate the transvalvular peak velocity. Some limitations can be addressed with clinical expertise in CMR, which is predominantly limited to cardiac territory centres. As previously noted, while 4D flow CMR can mitigate some of the constraints inherent in 2D flow, it introduces its unique limitations that are not associated with 2D flow.^39^ Four-dimensional flow CMR currently mandates long acquisition and post-processing time. Ideally, due to the higher under-sampling methods used for acceleration, 4D flow is best done after gadolinium contrast injection. Non-contrast 4D flow can significantly underestimate flows and peak velocities. A further complication with 4D flow is that numerous software solutions currently lack suitable valve tracking and dedicated MV assessment capabilities. Although these solutions are in the process of development, their widespread availability remains limited.

## Specific scenarios

### Transcatheter structural MV interventions

In the realm of transcatheter structural MV interventions, CMR is less helpful in establishing precise mitral path-anatomy but has a potential clinical role in grading the severity of MR—both pre and post-intervention, determining haemodynamic implications and evaluating ventricular remodelling—an essential prognostic factor. In more recent work, Ricci *et al*.^58^ have published reference values of mitral annular dimensions using 5065 consecutive UK Biobank participants’ CMR data field. One of their key findings was that the reproducibility of MV annular dimensions was excellent. The authors noted that the reference values will pave the way to improve the distinction between normal and pathological states in CMR examination, prompting the identification of subjects that may benefit from advanced cardiac imaging for annular sizing and planning of valvular interventions.

Post-MitraClip implantation, quantitative assessment of residual MR by transthoracic echocardiography remains challenging due to multiple eccentric jets and artefacts from the clips. The MitraClip device is compatible with CMR and can be safely visualized using CMR. Importantly, a study by Radunski *et al*.^59^ observed that the assessment of endocardial contours was not compromised by the MitraClip device-related artefact. Their study concluded that CMR was able to map longitudinal changes in LV and LA volumes pre and post-MitraClip insertion and percutaneous MitraClip repair results in predominantly reverse LV remodelling. In another study by Hamilton-Craig *et al*.,^60^ where they recruited 25 patients who went on to have percutaneous MitraClip, only 16 patients were able to have CMR. The main reasons for exclusion were arrhythmia and non-compatible devices. Nevertheless, in patients who were suitable for the scan, they showed that CMR performed more consistently and precisely for the quantification of MR post-MitraClip insertion than echocardiography. Furthermore, a study by Velu *et al*.^61^ where patients received CMR scans pre MitraClip concluded that the presence of myocardial fibrosis on LGE was predictive of adverse outcomes at one month. They showed that 69% of patients with myocardial fibrosis experienced adverse outcomes, contrasting with only 11% of patients without myocardial fibrosis (*P* = .01). In the case of mitral transcatheter edge-to-edge repair (TEER), a study demonstrated the value of native T1 assessment for better prognostication of patients with HF with secondary MR following TEER.^62^ These studies highlight that both echocardiography and CMR are complementary imaging modalities with their specific pros and cons, and in real-world scenarios, they should be used in conjunction to better manage patients with MV diseases.

### Arrhythmic mitral valve prolapse

In the past decade, cumulating evidence pointed out how a subset of patients with MVP have an increased risk of sustained ventricular tachycardia (VT) and sudden cardiac death, referred to as malignant MVP phenotype.^63^ In this setting, CMR has the fundamental role of highlighting the presence of ‘high-risk’ features, such as the presence of mitral annular disjunction (MAD) or myocardial fibrosis in the left ventricle.^64^ Mitral annular disjunction is the distance that is measured from the LA wall–MV leaflet junction to the top of the LV wall at end-systole in long-axis cines, and it is defined as present if ≥1.0 mm.^65^ Transthoracic echocardiography is the first-line examination to detect the presence of MAD; however, sometimes, it may be challenging and more easily assessed by CMR.^65^ The role of MAD in arrhythmogenesis has been largely evaluated in different retrospective studies, which have shown controversial results. On the one hand, it seems that its presence *per se* may be associated with a higher incidence of ventricular arrhythmias.^66^ Recent studies have highlighted how this feature can also be easily found even in normal hearts.^67,68^ If more in-depth studies are needed to depict its effective role in arrhythmogenesis, to date, MAD remains one of the high-risk arrhythmic features in patients with MVP. Nevertheless, the presence of myocardial fibrosis in the left ventricle can be detected only with CMR. Firstly described in the landmark paper of Basso *et al*.^69^ and typically evaluated in LGE sequences, it is traditionally located in the inferior, infero-lateral wall, and at the tip of papillary muscles.^70^ The strong link between myocardial fibrosis and arrhythmogenesis has been confirmed thereafter in several studies,^70,71^ suggesting how the presence of LGE improves the prediction of adverse events beyond prolapse severity and prior ventricular arrhythmia^71^ and its evaluation is strongly recommended in all patients with the suspicion of malignant MVP phenotype.^72^ The fact that MVP may influence LV remodelling has been recently investigated using novel CMR techniques such as T1 mapping^73,74^ or CMR feature tracking.^75,76^ However, if the presence of interstitial fibrosis or a higher degree of myocardial deformation seems to be associated with MVP, they are not routinely used in clinical practice as more in-depth studies are needed to evaluate their potential role in arrhythmic risk stratification.

### MS

Even though the evidence of CMR in MS is not as established as for MR assessment, CMR could still emerge as a potential tool for MS assessment. It provides a clear visualization of the degree of stenosis and allows for accurate measurement of the valve area, even with angulated outflow tracts. Cardiovascular magnetic resonance can also assess the haemodynamic severity of the stenosis through velocity mapping. However, accuracy may be reduced at higher velocities due to signal loss from turbulence and phase shift errors.

Studies have shown a high agreement between CMR and echocardiography in assessing MV area before and after percutaneous balloon mitral valvuloplasty, a common intervention for MS.^77,78,79^ Echocardiography can underestimate LA volume compared with CMR, and for longitudinal monitoring of LA and LV remodelling pre-/post-MV intervention, CMR is preferred to assess the degree of remodelling.^78^ A study which investigated the effects of rheumatic MS on chamber remodelling in the endemic zones showed that these patients have biventricular dysfunction, right and LA remodelling, and scar/fibrosis at the RV insertion sites. Furthermore, there is evidence that myocardial fibrosis is associated with post-operative morbidity after MV surgery in patients with rheumatic MS.^80^

**Figure 9:**
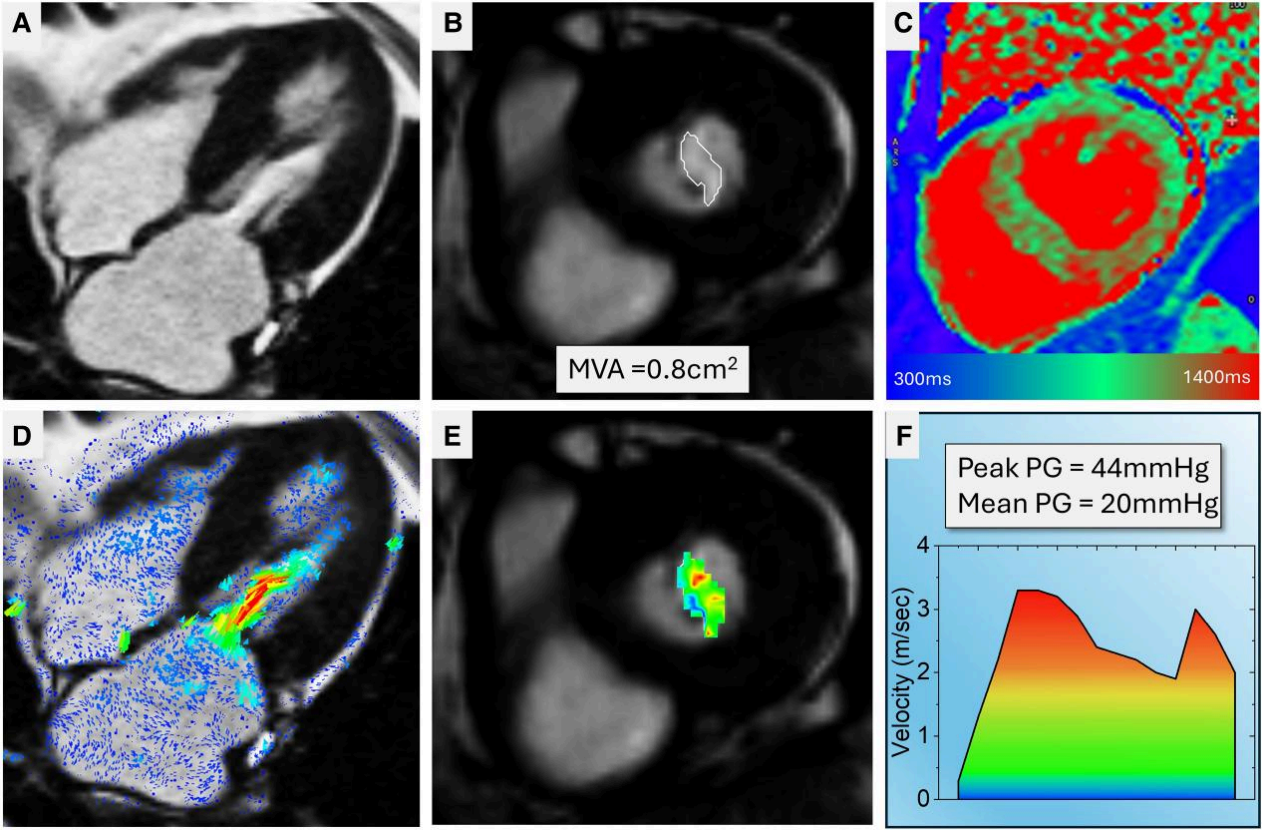
MS assessment by CMR. (*A* and *B*) Cine images demonstrate restrictive mitral valve opening with flow acceleration seen as a de-phasing artefact through the papillary muscles. On the short-axis, cut-through at the narrowest opening during peak mitral inflow, the mitral valve area on planimetry was .8 cm^2^, consistent with severe MS. Furthermore, the left atrium is significantly dilated again, suggesting raised LA pressures. (*C*) Native T1 values were higher than normal in the septum suggestive of myocardial changes. (*D* and *E*) Superimposed velocity vector using 4D flow displayed on top of cine images demonstrates the spatial location of the peak inflow velocities. (*F*) Quantification of mitral inflow velocities using 4D flow at the level of maximum velocities in the log-axis observed in *D*. The mean pressure gradient across the mitral valve was 20 mmHg—consistent with severe MS

Cardiovascular magnetic resonance assessment of MS is done routinely by two-, three-, and four-chamber as well as short-axis cines. The short-axis cines should be planned parallel to the mitral annular plane with a slice thickness of 5 mm and preferably without any gap to allow capturing true MV tips for MV area calculation by planimetry. The long-axis cines allow for a qualitative assessment of the MV mobility, its opening and closure, and any issues with the sub-valvular apparatus for an aetiological assessment of MS. On short-axis cines, a free-hand region of interest is drawn at the maximal opening of the MV in diastole to measure MV area. This method is robust, simple, and consistent with both transthoracic and transoesophageal assessment of MV area.^78^ In the context of assessing transvalvular peak velocity at the MV tips, the use of 2D flow is not advised. This method tends to underestimate the peak velocity significantly, as unlike continuous-wave Doppler, 2D flow lacks the capability to record all velocities in a longitudinal line. Four-dimensional flow CMR may be able to solve the issue with 2D flow as it can account for valve motion when assessing the peak velocity at the tips of MV, which can be done in an automated way using three-dimensional velocity information through the MV^81,82^ (*Figure 9*).

## Conclusion

Cardiovascular magnetic resonance routinely complements echocardiography in MR quantification, patient risk stratification based on LA and LV remodelling, scar/fibrosis burden, and arrhythmic risk assessment. It is endorsed as a Class I indication for establishing the aetiology and prognosis of various cardiomyopathies often associated with MR.^12^ Integrating CMR is recommended to enhance the diagnosis and management of MV disease and related cardiomyopathies.

## Supplementary data

Supplementary data are available at *European Heart Journal* online.

## Supplementary Material

ehae801_Supplementary_Data

## Declarations

### Data Availability

No data were generated or analysed for or in support of this paper.
