# Supplementary material for: Cardiovascular magnetic resonance imaging in mitral valve disease
Source: Eur Heart J. 2024 Nov 20;46(7):606–19. doi: 10.1093/eurheartj/ehae801 (PMC11825178; doi:10.1093/eurheartj/ehae801)
Supplement: ehae801_Supplementary_Data [file ehae801_supplementary_data.zip › Supplementary Section Videos.docx]

**Videos**

**Video 1.** A walk-through tutorial on how to quantify mitral regurgitation using standard techniques is described.

**Video 2.** A walk-through tutorial on how to quantify mitral regurgitation using four-dimensional flow CMR techniques and how to apply conservation of mass principle to improve confidence and precision in MR reporting.
